# Diverse associations between adiposity and blood pressure among 80,000 multi-ethnic Chinese adults

**DOI:** 10.1186/s12889-023-15224-7

**Published:** 2023-02-09

**Authors:** Jiayi Chen, Haojiang Zuo, Xinyu Wu, Yuan Zhang, Qiang Tan, Zhimiao Yu, Ciren Laba, Yongyue Pan, Jianzhong Yin, Feng Hong, Peibin Zeng, Xing Zhao

**Affiliations:** 1 West China School of Public Health and West China Fourth Hospital, Sichuan University, 16#, Section 3, Renmin Road South, 610041 Chengdu, Sichuan China; 2 Chongqing Center for Disease Control and Prevention, Chongqing, China; 3 Chengdu Center for Disease Control and Prevention, Chengdu, Sichuan China; 4 Tibet Center for Disease Control and Prevention, Lhasa, Tibet China; 5 Tibet University, Lhasa, Tibet China; 6 School of Public Health, Kunming Medical University, Kunming, Yunnan China; 7 Baoshan College of Traditional Chinese Medicine, Baoshan, Yunnan China; 8 School of Public Health, the Key Laboratory of Environmental Pollution Monitoring and Disease Control, Guizhou Medical University, Guiyang, Guizhou China

**Keywords:** Blood pressure, Central adiposity, General adiposity, Multi-ethnic region, Population characteristics

## Abstract

**Background:**

Adiposity is widely recognized as one of the risk factors for high blood pressure (BP) and increasing adiposity is associated with elevated BP. However, which measures of adiposity could be most strongly associated with BP in multi-ethnic population remains uncertain, giving rise to implications that population-based adiposity measures could be necessary.

**Methods:**

80,000 multi-ethnic adults recruited from 5 provinces across Southwest China during 2018 ~ 2019 were studied. Multiple linear regression was applied to investigate the associations of systolic blood pressure (SBP) with: (1) two measures of general adiposity, body mass index (BMI) and height-adjusted weight; and (2) three measures of central adiposity, waist circumference (WC), hip circumference (HC) and waist hip ratio (WHR).

**Results:**

Two distinct population-specific patterns were identified, as “BMI to SBP” and “WC to SBP”. 90% of the participants fall into “BMI to SBP” pattern, in which the associations of SBP with BMI were independent of WC, and SBP-WC associations were considerably decreased by adjustment for BMI. And in this pattern, 10 kg/m^2^ greater BMI was associated with 11.9 mm Hg higher SBP on average. As for the rest population (Han males in Yunnan and Tibetans in Lhasa), they are suited for “WC to SBP” pattern, 10 cm wider WC was associated with 3.4 mm Hg higher SBP.

**Conclusion:**

Our results indicated that when selecting proper predictors for BP, population-specific adiposity measures are needed, considering ethnicity, sex and residing regions. A better understanding of adiposity and BP may better contribute to the potential clinical practices and developing precision application strategies.

**Supplementary Information:**

The online version contains supplementary material available at 10.1186/s12889-023-15224-7.

## Introduction

High blood pressure is the leading global risk factor for cardiovascular diseases, which result in substantial morbidity and mortality worldwide [1–3]. One of the key determinants of blood pressure is adiposity [4, 5], that is commonly classified into general adiposity and central adiposity. Previous publications have drawn inconsistent conclusions that either general or central adiposity could be most strongly associated with blood pressure (BP) [6–8]. For instance, some reported that the general adiposity variables (e.g. BMI) were more strongly associated with BP than central adiposity ones in Chinese Han ethnicity, Japanese and Western population [9–13]. On the contrary, others described central adiposity variables (e.g. WC) had stronger relation to BP [14–16]. In addition, the strength of associations also differed. A study on 0.5 million Chinese Han ethnicity indicated that systolic blood pressure (SBP) increased by 16 mm Hg for each 10 kg/m^2^ higher BMI, which was about 50% greater than that observed in the Western population [11, 17], implying that population-specific adiposity thresholds is needed. Likewise, whether these conclusions could be applied to multi-ethnic population remains uncertain, when ethnicity is increasingly considered as an important risk factor for non-communicable diseases throughout the world [18].

There are 56 different ethnic groups residing in China, and southwest China is home to one-third of minority people covering 55 ethnic minority groups. Previous studies primarily focused on the associations between adiposity and BP at regional or national level [11, 12, 17], but with discordant conclusions and with little evidence regarding multi-ethnic population. These questions are particularly relevant in multi-ethnic areas, where ethnic characteristics could confound the strength of these associations. Therefore, a better understanding of adiposity and BP is needed, so as to contribute to the potential clinical practices when applying adiposity markers as predictors of BP in multi-ethnic population. Here, we conducted a cross-sectional study to investigate the associations between adiposity and BP among multi-ethnic population in Southwest China.

## Methods

### Study population

The design and conduct of the China Multi-Ethnic Cohort (CMEC) study has been reported elsewhere [19]. Between May 2018 and September 2019, a total of 99 556 participants (men: n = 39,793 and women: n = 59,763) mostly aged from 30 to 79 years were recruited from six ethnicities: Han, Tibetan (aged from 18 to 79 years), Yi, Miao, Bai, Bouyei, and Dong from five provinces of Southwest China (Sichuan, Chongqing, Tibet, Yunnan, and Guizhou). Based on different ethnics and residing areas, nine populations were categorized, previously described as: Han ethnic in Basin (Sichuan and Chongqing province), Han ethnic in Yunnan, Tibetans in Lhasa, Tibetans in Aba-Sichuan and other five ethic-specific population consisting of Yi, Miao, Bai, Bouyei and Dong. Baseline data collection included a computerized questionnaire, physical examinations and collection of blood samples. The study was approved by the ethics committees of Sichuan University (No: K2016038 and K2020022) and written informed consent was obtained from all participants.

### Adiposity and blood pressure measures

In this study, five main adiposity variables (measured or derived) were obtained, including two general adiposity variables (height-adjusted weight and BMI) and three central adiposity variables (WC, HC and WHR). Calibrated scales, fixed stadiometer and non-stretchable tapes were used and all anthropometric indexes (wearing light clothes) were measured by trained personnel. Weight, standing height, WC, and hip circumference (HC) were measured to the nearest 0.1 kg or 0.1 cm, respectively. WC was measured at the midpoint between the iliac crest and the lower rib, and HC was measured at the maximum circumference around the buttocks. BMI was calculated as weight (Kg) divided by the square of height (m^2^) and WHR was the ratio of WC to HC.

The BP measurements of study participants were taken by trained medical personnel using electronic sphygmomanometers (OMRON Corporation), which were calibrated before measurements. All BP measurements were performed in a seated, upright position three times and the mean values of the three measures were used to calculate SBP and diastolic blood pressure (DBP). Previous studies have reported that SBP is a better predictor of vascular mortality than DBP [20]. And the associations of adiposity with blood pressure are more evident with respect to SBP than DBP [11, 21], which was also observed in our previous investigation. Therefore, main results are based on SBP in our study.

### Blood biochemistry and covariates

Blood samples were collected from CMEC participants after overnight fasting and were transported by commercial courier to Third-Party Medical Laboratories where they were processed by standard biochemical techniques. Fasting serum glucose levels were determined by the Hexokinase method (Beckman). Total cholesterol (TC), high-density lipoprotein cholesterol (HDL-C) and low-density lipoprotein cholesterol (LDL-C) levels were evaluated by using the enzyme colorimetry (Beckman). Triglyceride (TG) levels were measured by using glycerol phosphate oxidase - peroxidase method reagent (Beckman).

We ascertained dyslipidemia according to the guideline for the management of dyslipidemia of adults in China [22], as defined as TC ≥ 6.2 mmol/L, or TG ≥ 2.3 mmol/L, or LDL-C ≥ 4.1 mmol/L. Decreased HDL-C was defined as HDL-C < 1.0 mmol/L. Presence of diabetes mellitus (DM) was based on self-reported physician diagnosis, or a fasting glucose value ≥ 7.0 mmol/L or HbA1c ≥ 6.5%, according to American Diabetes Association criteria [23].

Smoking was defined as at least smoking 100 cigarettes till the investigation. We divided smoking into three categories: never, current regular, ex-regular. At the same time, we divided alcohol consumption into five categories: non-drinker, occasional, seasonal, monthly, weekly. We used the metabolic equivalent tasks (MET) index to describe physical activity. Vegetables, red and processed meat, oil (vegetable) and salt were defined as average intake per person per week.

### Statistical analysis

**Fig. 1:**
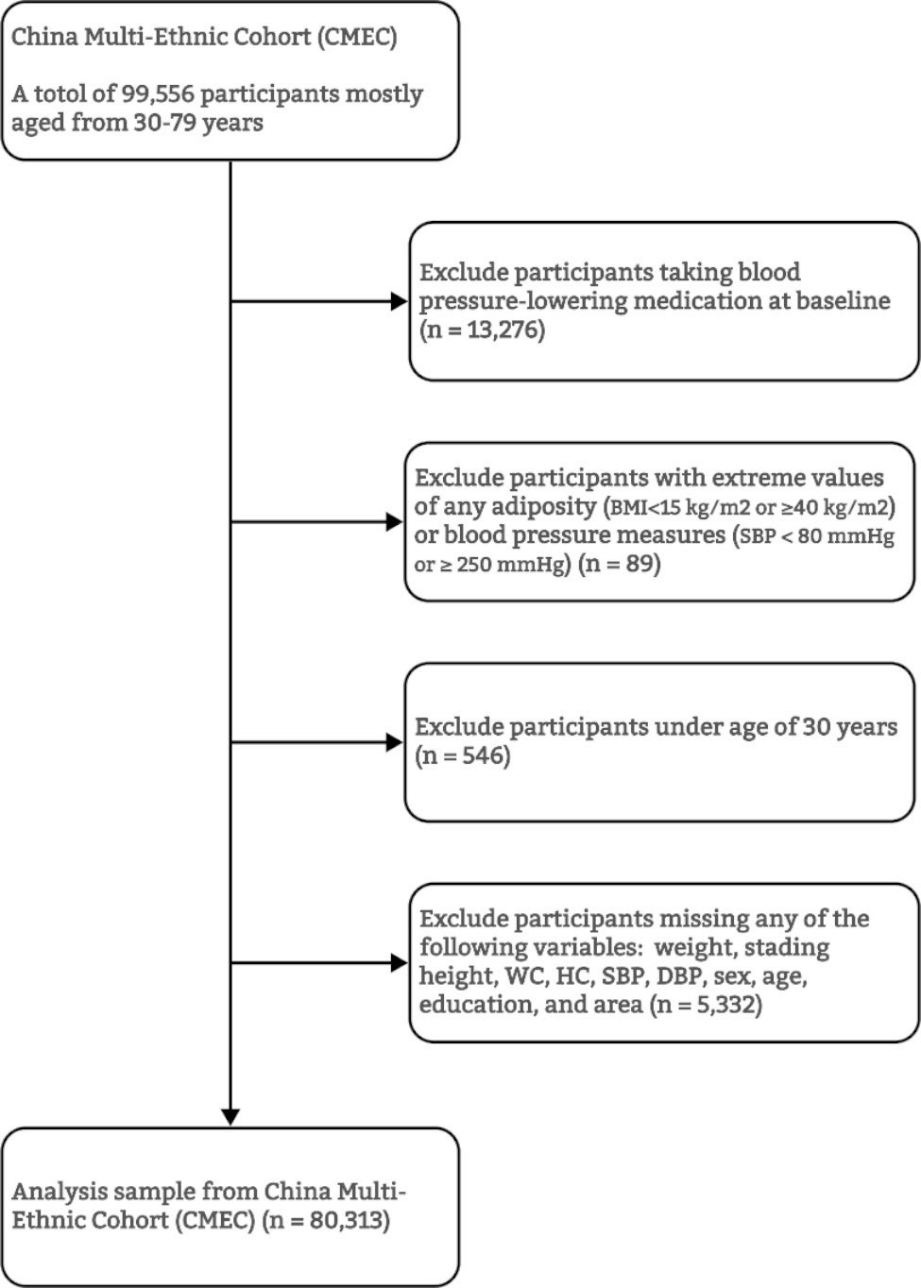
Flow chart of the analysis sample in the study. WC: waist circumference, HC: hip circumference

In this study, participants taking blood pressure-lowering medication at baseline were excluded (n = 13,276). In addition, individuals with extreme values of any adiposity or blood pressure measures (n = 89, e.g. BMI < 15 kg/m^2^ or ≥ 40 kg/m^2^; or SBP < 80 mmHg or ≥ 250 mmHg), under age of 30 years (n = 546, all those were Tibetans), and missing data (n = 5332) were also excluded. After exclusions, 80,313 (80%) participants remained for further analyses (Fig. 1).

The Pearson partial correlation coefficient was used to calculate the sex-specific correlations between different adiposity and blood pressure variables, adjusted for the baseline age (5 categories) and area. Two regression strategies were implemented, including “categorical” analysis and continuous analysis. The continuous analysis treated two outcomes (BMI, WC) as numerical variables, while “categorical” analysis first categorized five outcomes (adiposity measures) into deciles, and then they were included into the model as numeric variables.

For “categorical” analysis, multiple linear regression was applied to calculate the adjusted SBP for each sex-specific decile of adiposity measures, with adjustment for age (continuous), education (6 categories: no formal school, primary school, middle school, high school, junior college, university or higher), area (6 categories: Sichuan, Chongqing, Guizhou, Yunnan, Tibet, Aba), WC (continuous, only for central adiposity), BMI (continuous, for general adiposity). The height-adjusted weight was obtained by regression. Adjusted SBP for each decile was plotted on the Y-axis, against the average adiposity for each decile on the X-axis. A straight line was fitted using inverse variance-weighted least squares method.

For continuous analysis, multiple linear regression (adjusting for same covariates as “categorical” analysis) was used to calculate the mean difference in SBP associated to 1SD higher level of adiposity measures.

Separate regressions within each level of the possible effect modifier were conducted to assess the effect modification in terms of continuous BMI and WC. All analyses were carried out using R version 4.0.3 and visualized via R package corrplot.

## Results

### General features of demographic, anthropometric measures, blood pressures and covariates

The mean (± SD) levels of each index of demographic, anthropometric measures, blood pressures and covariates, stratified by sex, were displayed in Table 1. Among the participants included, the overall mean age was 50.21 (11.12) years. On average, mean height was 164.54 (6.6) cm in men and 153.98 (6.1) cm in women, and mean weight was 65.92 (10.6) kg in men and 56.49 (8.8) kg in women. For general adiposity indices, the means BMI and height-adjusted weight was 24.01 (3.38) kg/m^2^ and 60.19 (18.13) kg, respectively, while the means of central adiposity measures for WC, HC and WHR were 82.01 (10.18) cm, 93.06 (7.16) cm and 0.88 (0.07), respectively. For blood pressure, the means of SBP and DBP were 123.18 (17.44) and 78.13 (10.68) mm Hg. All these 7 anthropometric measures in men were significantly higher than those in women. In addition, men were more likely to be smokers, drinkers and have more physical activities, and consume more meat, vegetables, salt and oil (vegetable) than women.

**Table 1:** General features of demographic, anthropometric measures, blood pressures and covariates on study participants

	Study participants (n = 80,313)	Male (n = 31,515)	Female (n = 48,798)	P value
Age, years	50.2 (11.1)	51.0 (11.4)	49.7 (10.9)	
Age distribution (years), n (%)				< 0.001
30–39	14,935 (18.6)	5511 (17.5)	9424 (19.3)	
40–49	26,031 (32.4)	9793 (31.1)	16,238 (33.3)	
50–59	21,909 (27.3)	8271 (26.2)	13,638 (27.9)	
60–69	12,852 (16.0)	5788 (18.4)	7064 (14.5)	
70–79	4586 (5.7)	2152 (6.8)	2434 (5.0)	
Anthropometric measures				
Height, cm	158.12 (8.1)	164.54 (6.6)	153.98 (6.1)	< 0.001
Weight, kg	60.19 (10.59)	65.92 (10.56)	56.49 (8.81)	< 0.001
Height-adjusted weigh, kg	60.19 (8.50)	60.93 (9.06)	59.71 (8.08)	< 0.001
BMI, kg/m^2^	24.01 (3.38)	24.31 (3.33)	23.81 (3.40)	< 0.001
WC, cm	82.01 (10.18)	84.92 (9.84)	80.14 (9.95)	< 0.001
HC, cm	93.06 (7.16)	93.81 (6.88)	92.57 (7.29)	< 0.001
Waist-hip ratio	0.88 (0.07)	0.90 (0.06)	0.86 (0.07)	< 0.001
Blood pressure				
SBP, mmHg	123.18 (17.45)	126.61 (16.75)	120.96 (17.53)	< 0.001
DBP, mmHg	78.13 (10.68)	81.17 (10.79)	76.17 (10.13)	< 0.001
Initial nine populations, n (%)				< 0.001
Han ethnic in Basin	37,810 (47.1)	17,075 (54.2)	20,735 (42.5)	
Han ethnic in Yunnan	8613 (10.7)	3048 (9.7)	5565 (11.4)	
Tibetans in Lhasa	4712 (5.9)	1825 (5.8)	2887 (5.9)	
Tibetans in Aba-Sichuan	4363 (5.4)	1626 (5.2)	2737 (5.6)	
Yi ethnic	5117 (6.4)	1686 (5.3)	3431 (7.0)	
Miao ethnic	4347 (5.4)	1542 (4.9)	2805 (5.7)	
Bai ethnic	5022 (6.3)	1452 (4.6)	3570 (7.3)	
Bouyei ethnic	4836 (6.0)	1387 (4.4)	3449 (7.1)	
Dong ethnic	5493 (6.8)	1874 (5.9)	3619 (7.4)	
Meat consumption, g/week	668.64 (643.82)	787.62 (701.90)	591.79 (590.75)	< 0.001
Vegetable consumption, g/week	2164.27 (1455.92)	2199.80(1499.45)	2141.33 (1426.65)	< 0.001
Oil plant consumption, g/week	295.31 (208.83)	305.32 (214.53)	288.84 (204.82)	< 0.001
Salt consumption, g/week	46.65 (28.80)	47.20 (29.13)	46.29 (28.59)	< 0.001
Smoking				< 0.001
Never	60,486 (75.3)	12,440 (39.5)	48,046 (98.5)	
Current regular	16,153 (20.1)	15,522 (49.3)	631 (1.3)	
Ex-regular	3660 (4.6)	3546 (11.3)	114 (0.2)	
Alcohol consumption				< 0.001
Non-drinker	45,261 (56.4)	10,666 (33.9)	34,595 (70.9)	
Occasional	20,993 (26.1)	9440 (30.0)	11,553 (23.7)	
seasonal	895 (1.1)	488 (1.5)	407 (0.8)	
Monthly	2844 (3.5)	1931 (6.1)	913 (1.9)	
Weekly	10,294 (12.8)	8980 (28.5)	1314 (2.7)	
Physical activity METs/day	33.09 (17.95)	33.30 (18.27)	32.96 (17.74)	0.009
Diabetes mellitus				< 0.001
No	71,333 (91.7)	27,129 (89.0)	44,204 (93.5)	
Yes	6420 (8.3)	3358 (11.0)	3062 (6.5)	
Dyslipidemia				< 0.001
No	37,469 (46.7)	12,839 (40.7)	24,630 (50.5)	
Yes	41,312 (51.4)	18,063 (57.3)	23,249 (47.6)	
Reduced HDL-cholesterol				< 0.001
No	73,852 (92.0)	27,275 (86.5)	46,577 (95.4)	
Yes	6461 (8.0)	4240 (13.5)	2221 (4.6)	

### The age distribution and correlations of adiposity and blood pressures measures

Based on the pattern of associations between adiposity/blood pressure measures and age, the study participants were divided into three groups: Han ethnic in Yunnan (H-Y), Tibetans in Lhasa (T-L) and other seven ethics (O-S). SBP showed a strongly positive correlation with age (Figure S1). By contrast, DBP presented an inverted U-shape relationship with age, except for T-L, whose DBP increased monotonically with age. BMI also presented an inverted U-shape relationship with age in both groups. However, the associations of WC and age varied in different populations and sex. For T-L, WC slightly increased with age, both in men and women, while for the rest populations, WC marginally decreased in men and increased in women.

WC and BMI were highly correlated in most populations (H-Y and O-S) with coefficients ranging from 0.78 to 0.84 (Figure S2). However, for T-L, the correlation between adiposity measures and BMI was relatively weaker than the other two populations, with WC and BMI the highest correlation coefficients observed.

### Associations of adiposity and SBP

**Fig. 2:**
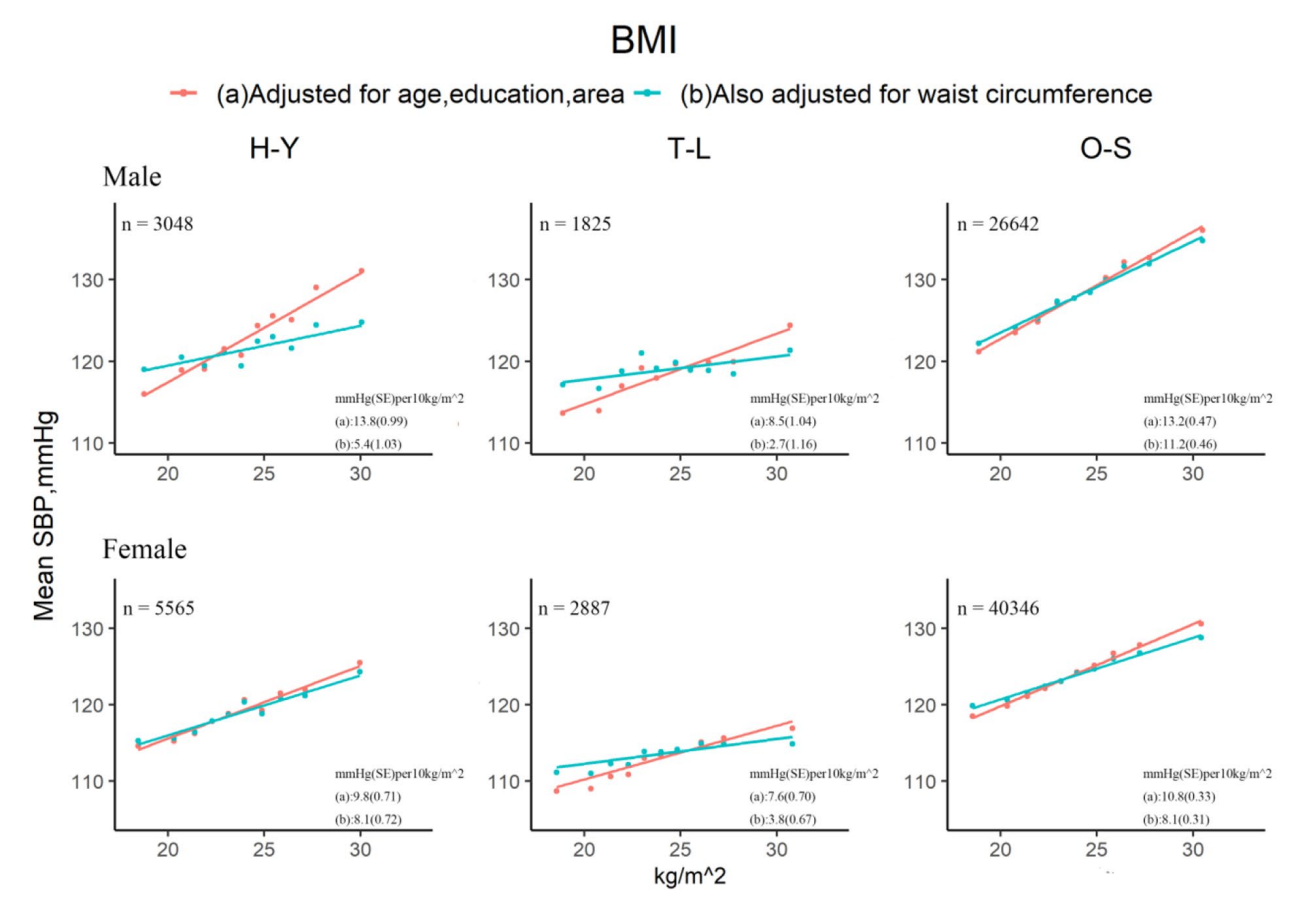
Sex-specific associations between BMI and SBP for the three populations, before and after adjustment for WC. The means of SBP were calculated for each sex-specific decile of BMI, with (a) adjustment for age, education and area; and (b) additional adjustment for waist circumference (as a continuous variable). H-Y: Han ethnic in Yunnan, T-L: Tibetans in Lhasa, O-S: other seven ethics

The sex-specific of BMI and SBP was positively correlated in three populations (Fig. 2). With basic adjustment (red line), each 10 kg/m^2^ higher BMI was associated with about 8.5 ~ 13.8 mm Hg higher SBP in men and 7.6 ~ 10.8 mm Hg higher SBP in women, varied among populations. Further adjustment for WC had small effect on the shape of these associations in O-S, H-Y (female), but largely reduced the magnitude of these associations in H-Y (male) by 61%, T-L by 68% (male) and 50% (female). The association of height-adjusted weight and SBP showed a similar pattern as that of BMI and SBP (Figure S3).

**Fig. 3:**
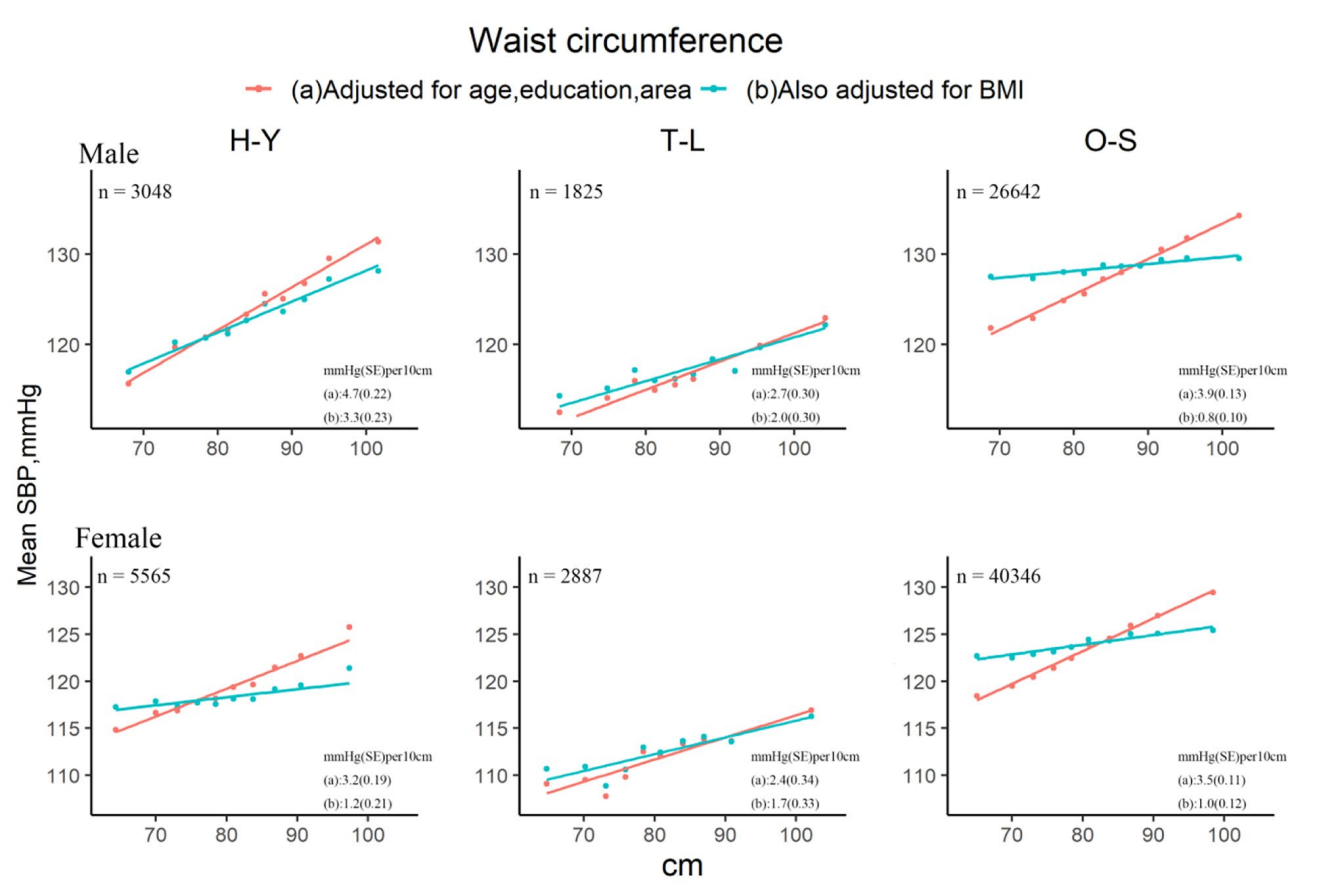
Sex-specific associations between WC and SBP for the three populations, before and after adjustment for BMI. The means of SBP were calculated for each sex-specific decile of WC, with (a) adjustment for age, education and area; and (b) additional adjustment for BMI (as a continuous variable). H-Y: Han ethnic in Yunnan, T-L: Tibetans in Lhasa, O-S: other seven ethics

All three markers of central adiposity, WC, HC and WHR, were also positively related to SBP. For WC, after basic adjustment, each 10 cm wider WC was associated with about 2.4 ~ 4.7 mmHg higher SBP (Fig. 3). The additional adjustment for BMI almost eliminated the associations between WC and SBP by 62.5% in H-Y (female) and O-S by 89% in men and 71% in women. Similar patterns were found in the relations of HC and WHR to SBP (Figure S4 and S5).

**Fig. 4:**
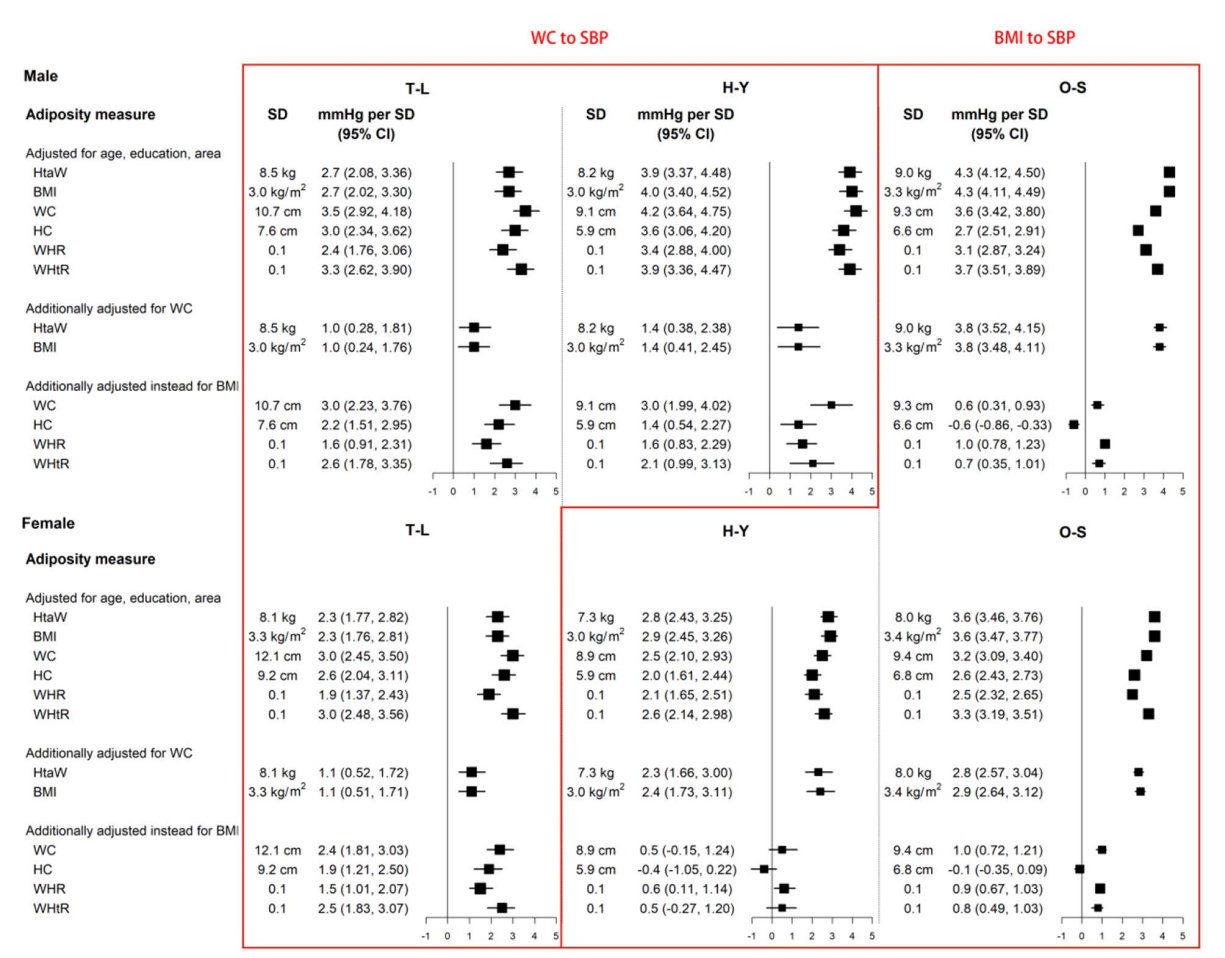
Higher SBP per standard deviation of each adiposity measure both in men and women. The differences in SBP per 1 SD of each adiposity measure were calculated, with SBP regressed on level of each adiposity measure as a continuous variable. Adjustment for covariates is as above. Closed squares represent the mean differences in SBP with size inversely proportional to the standerd deviation of the SBP. Horizontal lines represent the corresponding 95% CIs. H-Y: Han ethnic in Yunnan, T-L: Tibetans in Lhasa, O-S: other seven ethics

In addition, continuous analysis on associations between markers of adiposity and SBP was displayed as the difference in SBP per 1 SD higher level of adiposity (Fig. 4). The three populations (male and female) could be categorized into two patterns: BMI or WC, as suitable predictors of adiposity makers to SBP. The first was “BMI to SBP” pattern (H-Y (female) and O-S): BMI was the most significant predictor of SBP, with 1 SD increase in BMI corresponding to an increase of about 4.3 mmHg SBP in O-S (male), 2.9 mmHg SBP in H-Y (female), 3.6 mmHg SBP in O-S (female). And the associations were only slightly attenuated by additional adjustments for WC and HC.

H-Y (male) and T-L belonged to the other “WC to SBP” pattern. Within this pattern, WC was the strongest predictor after basic adjustment, with each 1 SD wider circumference associated with 3.5, 4.2 mmHg higher SBP in men, and 3.0 mmHg higher SBP in women.

**Fig. 5:**
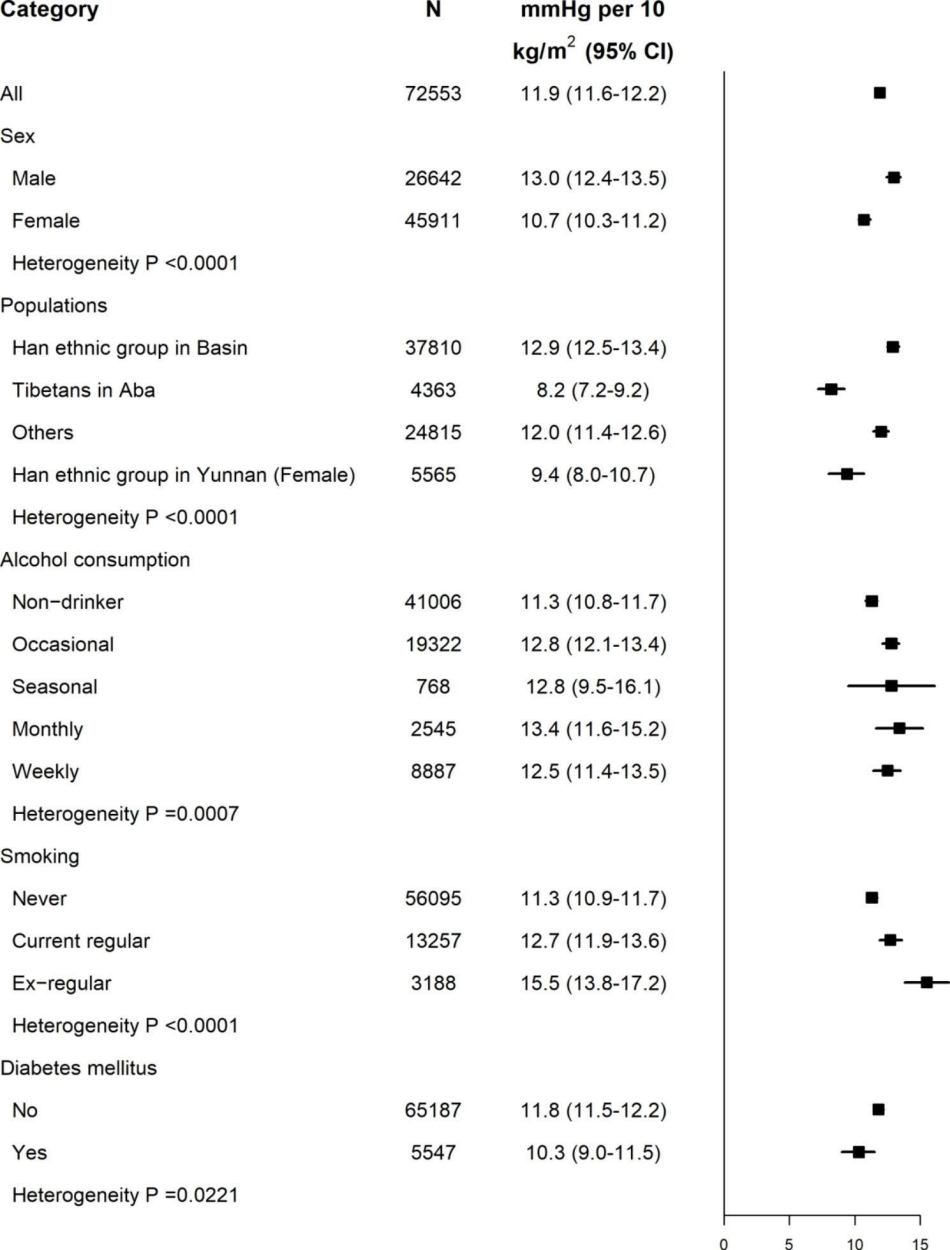
Higher SBP for each 10 kg/m^2^ BMI increase in different subgroups. Adjusted for age, area, education, and sex as appropriate

**Fig. 6:**
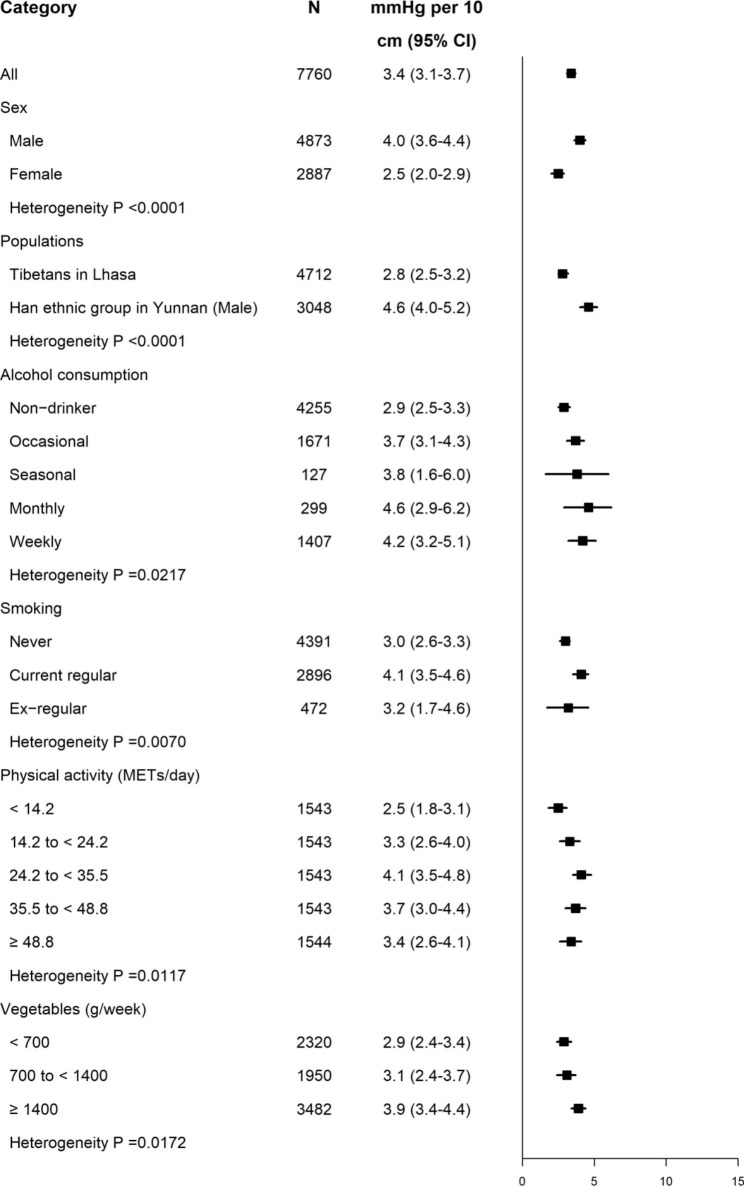
Higher SBP for each 10 cm WC increase in different subgroups. Adjusted for age, area, education, and sex as appropriate

BMI and WC, applied as the predictors of SBP, are both easy to implement in clinical practice. The strength of the associations of BMI (per 10 kg/m^2^) and WC (per 10 cm) with SBP, each in their suitable populations were presented in more detail, respectively (Figs. 5 and 6; Figure S6 and S7). For populations suited for the pattern of “BMI to SBP”, after adjusting the potential confounders, each 10 kg/m^2^ BMI higher was associated with 13 mmHg in men and 10.7 mmHg in women. Obvious variation in the strength of the association was observed in subgroups of smoking status, alcohol consumption and status of Diabetes mellitus.

For populations suited for the pattern of “WC to SBP”, each 10 cm WC higher was associated with 4.0 mmHg in men and 2.5 mmHg in women after adjusting the potential confounders. T-L and H-Y (Male) differed in the strength of associations at 2.8 mmHg and 4.6 mmHg, respectively.

## Discussion

In the present study, data of 80,313 multi-ethnic participants from Southwest China, were included to investigate the associations between different measures of adiposity and BP. Two distinct population-specific patterns of the associations were identified, between adiposity markers and BP. Consequently, among these participants, BMI or WC could be a proper predictor, and either could better be applied in specific populations regarding ethnicity and sex. 90% of the participants, including ethnics of Yi, Miao, Bai, Bouyei and Dong, Han ethnic in Basin, Tibetans in Aba-Sichuan and Han ethnic in Yunnan (female) suited for “BMI-SBP” pattern, and every 10 kg/m^2^ greater BMI was associated with about 11.9 mm Hg higher SBP. In populations including Han ethnic in Yunnan (male) and Tibetans in Lhasa that suited for “WC-SBP” pattern, 10 cm wider WC was associated with 3.4 mm Hg higher SBP.

It has been demonstrated that the associations of adiposity measures to SBP do vary in different races, and between sexes, despite the fact that they live in the same region. In our study, the majority of the participants fit for the pattern of “BMI to SBP”, in accord with findings from previous studies in Chinese (Han ethnicity), Japanese and western people [9–13]. On the other hand, participants of T-L and H-Y (male) fit for the pattern of “WC to SBP”, were consistent with those reports among western population [14–16]. Possible Reasons might be the combination of several factors such as dietary pattern, socioeconomic status, high-altitude habitation and population characteristics. However, further investigation is needed regarding this aspect. Furthermore, previous findings regarding the associations between sexes [6–8], were also found in H-Y population in this study. In general, the diverse patterns indicated that the selection of proper predictors of adiposity markers to BP should be stratified, regarding the confounders contributed by ethnicity, sex and residing regions, even in a certain part of a country.

The strength of associations, further in our study, also varied among different populations. For “BMI to SBP” suited population, the strength of the associations was comparable with previous research conducted in China (~ 4 mmHg SBP/ SD) [7, 11], but was stronger compared with those reported in Western populations [12, 17]. For population with “WC to SBP” pattern, the strength of associations (3 mmHg SBP/ SD in male T-L and H-Y) was stronger compared with that conducted in Greek EPIC cohort (2.26 mmHg/ SD) and that male 30–39 age group (2.26 mmHg/ SD) in India [21, 24].

The reasons for the diverse association of adiposity and SBP in ethnicity and sex are still not clear, which may partly be explained by variations in body fat distribution, antihypertensive treatment, sodium dietary intake and health status. Body fat distribution diversifies in different races [25]. For example, Asians are more likely to have a higher body fat content with lower BMI compared with Caucasians [26]. Besides, men tend to accumulate body fat in the upper body, whereas women in the lower body. In addition, available data suggest that high dietary sodium directly relates to progressive increase of SBP [27, 28]. Previous literature has described that the dietary sodium intake is higher in Chinese adults than many Western populations [29, 30], which may lead to the increasing strength of association in Chinese.

The relationship may not be universal, moreover, the mechanisms behind the association of adiposity with BP are not yet fully understood. There may be several pathways involved, such as increased sympathetic nervous system (SNS) activity, renin-angiotensin-aldosterone system (RAAS) dysfunction and adipokine dysregulation [31]. For example, perivascular adipose tissue (PVAT) inflammation dysfunction may play an important role in elevated peripheral resistance and blood pressure through elevating vascular tone and PVAT-derived adipokines, particularly adiponectin, leptin and resistin [32]. A study of 10 000 participants that underwent dual-energy x-ray absorptiometry imaging indicated visceral adipose tissue is the primary etiological component of excess adiposity [33], which allowed a detailed investigation underlying adiposity-related hypertension in a mostly white population. Thus, measures of body composition that directly quantify fat distribution in Chinese adults could potentially further uncover these variations and potential mechanisms.

This study has some limitations worth discussing. First, body fat percentage, which is also an indicator for general adiposity, was not measured in the baseline investigation. Meanwhile, common measures of adiposity are highly correlated, and may cause varying degrees of measurement error, which affects the strength of the associations. Thus, body composition measurements that directly quantify regional fat distribution are recommended in further research. Besides, some variables are self-reported, which may lead to recall bias.

In conclusion, diverse associations between adiposity and blood pressure were observed in this multi-ethnic study, and further stratified into two specific patterns with suited populations respectively. Our findings indicated that to better predict blood pressures, the selection of proper adiposity measures should be population-specified, taking ethnicity and other characteristics into consideration, and also shed light on important information to support such potential clinical practices in multi-ethnic areas.

## Electronic supplementary material

Below is the link to the electronic supplementary material.

Supplementary Material 1

## Data Availability

The datasets generated in the this study are not publicly accessible due to privacy and confidentiality reasons but are available from the corresponding author on reasonable request.

## Abbreviations

BP: Blood pressure
SBP: Systolic blood pressure
BMI: Body mass index
WC: Waist circumference
HC: Hip circumference
WHR: Waist hip ratio
CMEC: China Multi-Ethnic Cohort study
DBP: Diastolic blood pressure
TC: Total cholesterol
HDL-C: High-density lipoprotein cholesterol
LDL-C: Low-density lipoprotein cholesterol
FPG: Fasting plasma glucose
DM: Diabetes mellitus
MET: Metabolic equivalent tasks
H-Y: Han ethnic in Yunnan
T-L: Tibetans in Lhasa
O-S: Other seven ethics
