# Supplementary material for: Diverse associations between adiposity and blood pressure among 80,000 multi-ethnic Chinese adults
Source: BMC Public Health. 2023 Feb 9;23:298. doi: 10.1186/s12889-023-15224-7 (PMC9912499; doi:10.1186/s12889-023-15224-7)
Supplement: Supplementary file 1 — Supplementary Material 1 [file 12889_2023_15224_MOESM1_ESM.doc]

Supplemental Materials

A．


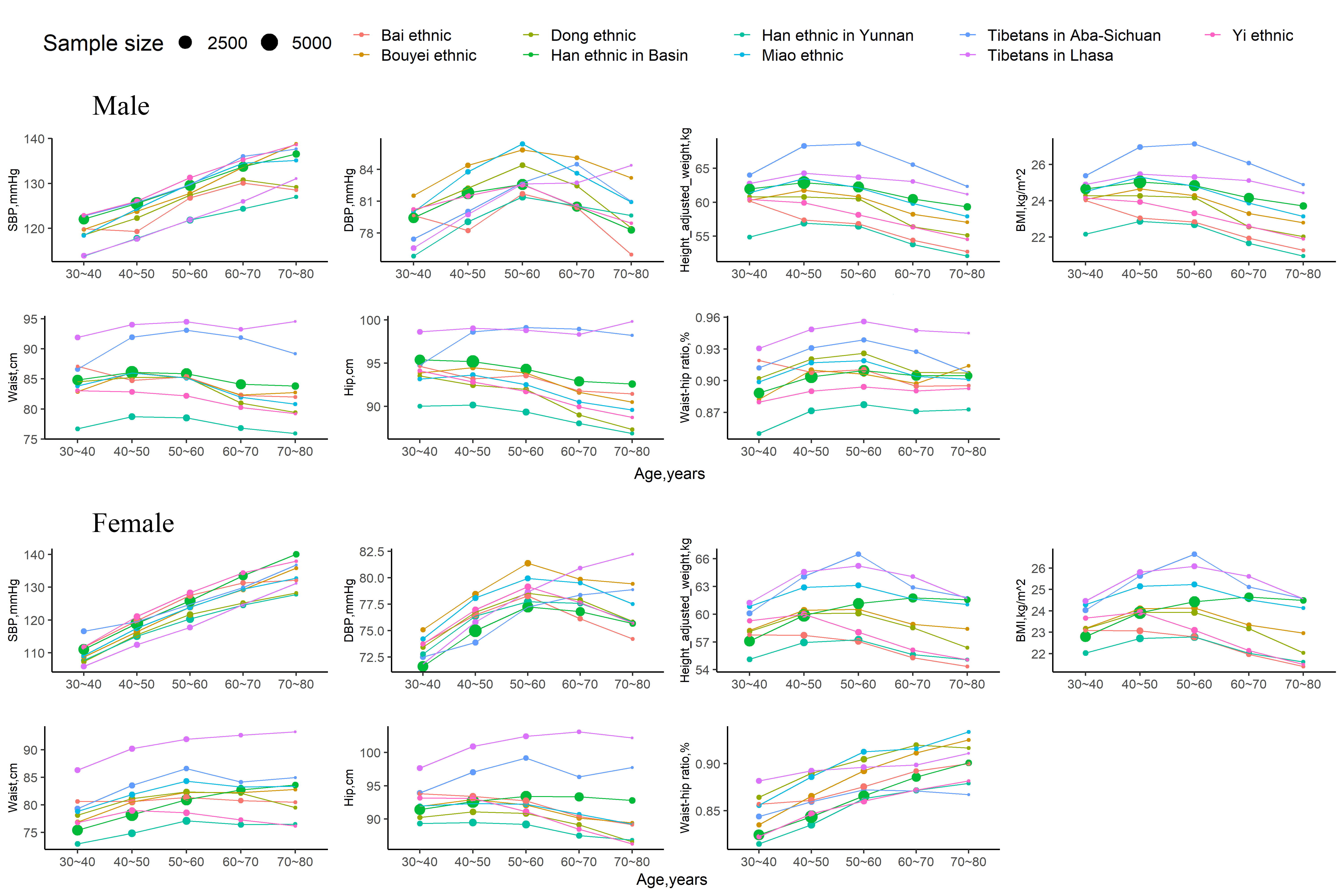


B．


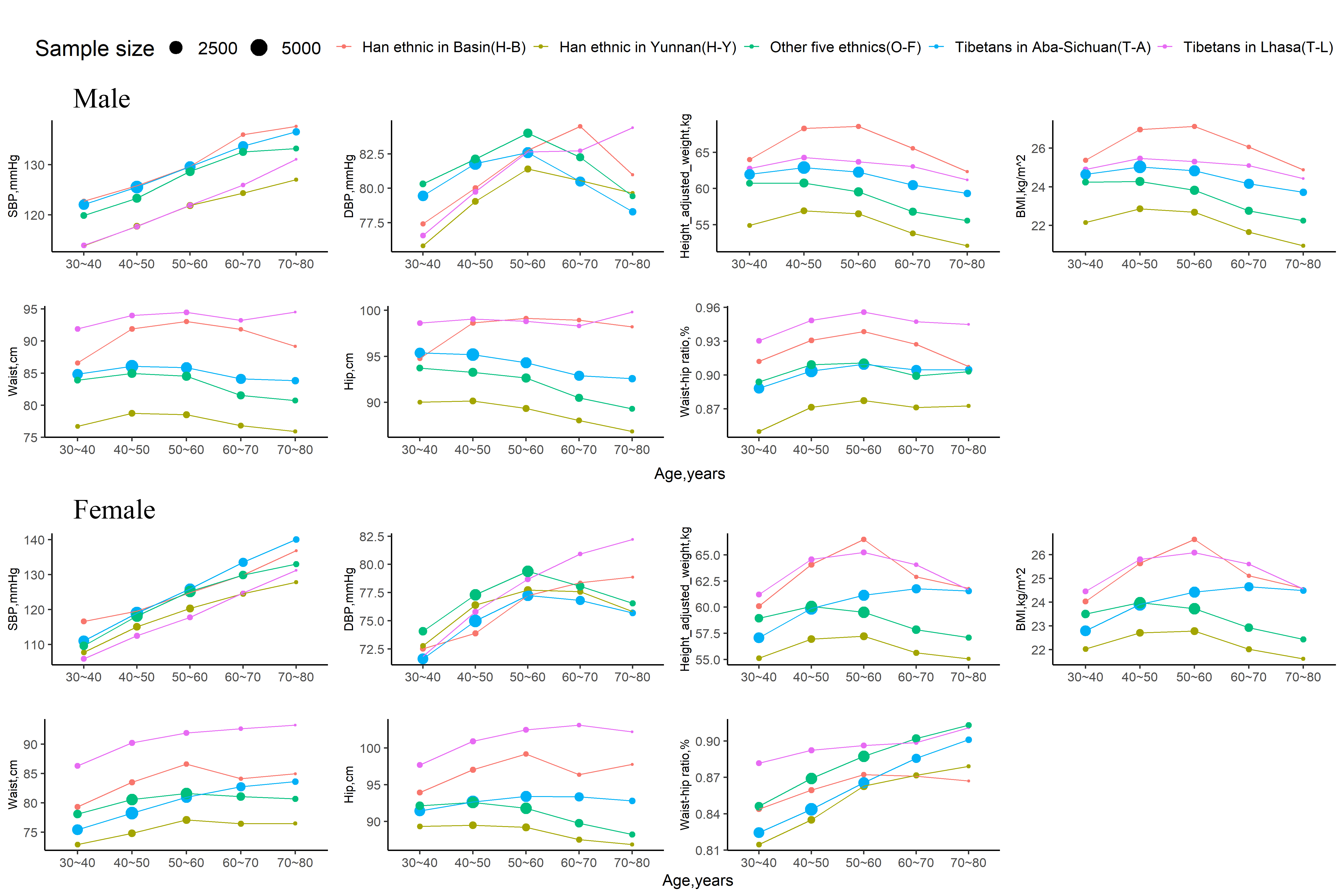


C．


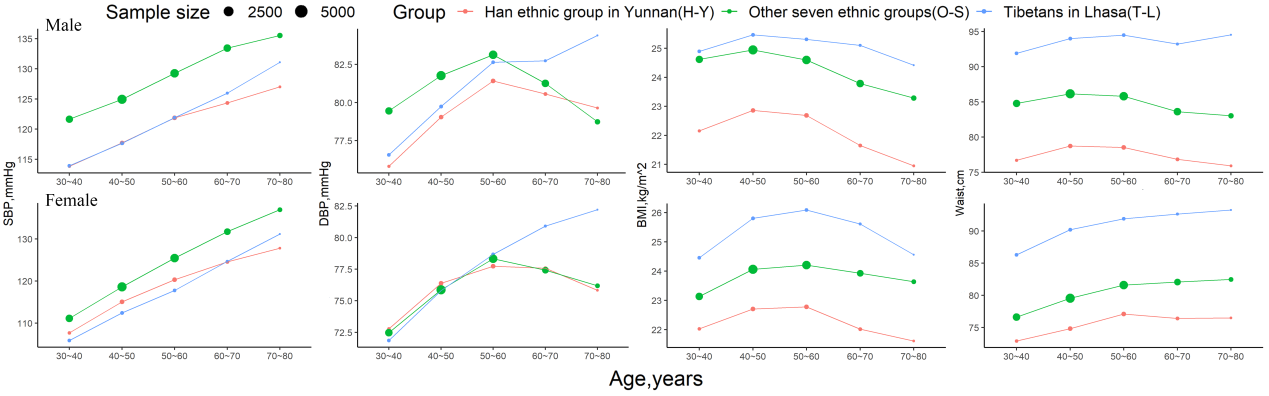
Figure S1. The age distribution of adiposity variables and blood pressures (SBP and DBP) for included populations. At first, the age distribution and correlations of adiposity and blood pressure measures were conducted in nine groups (Figure S1-A). As a result, ethnics of Yi, Miao, Bai, Bouyei and Dong shared a similar pattern on associations between adiposity/blood pressure measures and age. Therefore, these five ethnics were merged as O-F (other five ethics), along with the rest four groups (Han ethnic in Basin, H-B; Han ethnic in Yunnan, H-Y; Tibetans in Lhasa, T-L; and Tibetans in Aba-Sichuan, T-A) for further analysis (Figure S1-B). Furthermore, based on the pattern of associations between adiposity/blood pressure measures and age, the former five groups were further merged into three: Han ethnic in Yunnan (H-Y), Tibetans in Lhasa (T-L) and other seven ethics (O-S) (Figure S1-C).


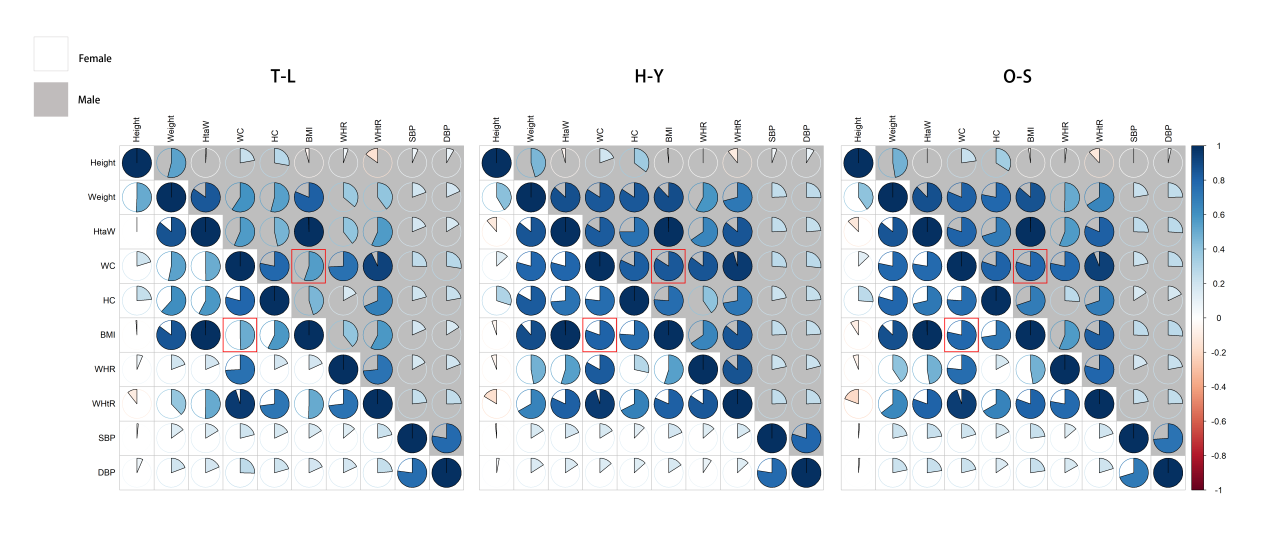


Figure S2. Correlations coefficients between adiposity indices and blood pressure for three populations.

H-Y: Han ethnic in Yunnan, T-L: Tibetans in Lhasa, O-S: other seven ethics


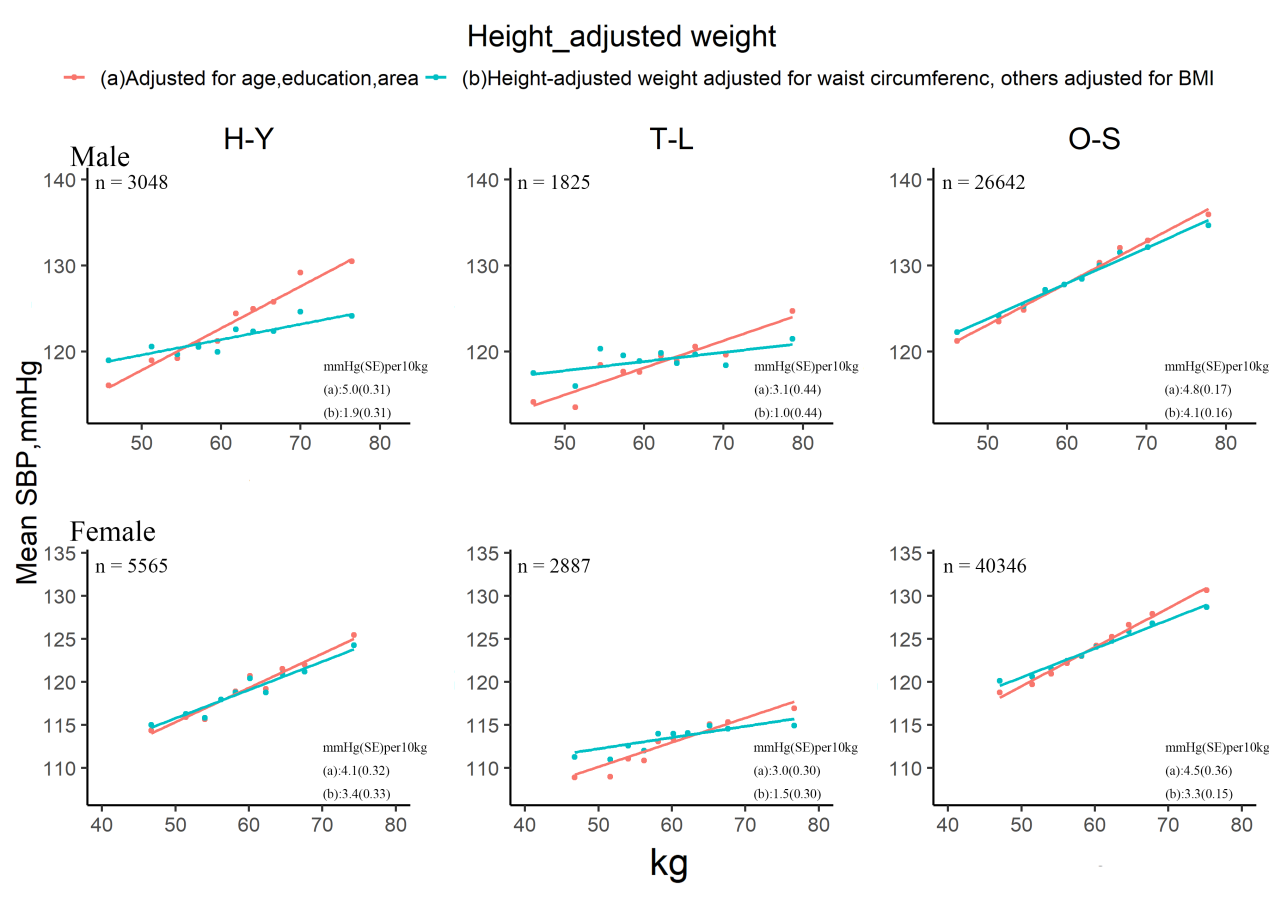


Figure S3. Sex-specific associations between height-adjusted weight and SBP for the three populations, before and after adjustment for WC.

H-Y: Han ethnic in Yunnan, T-L: Tibetans in Lhasa, O-S: other seven ethics


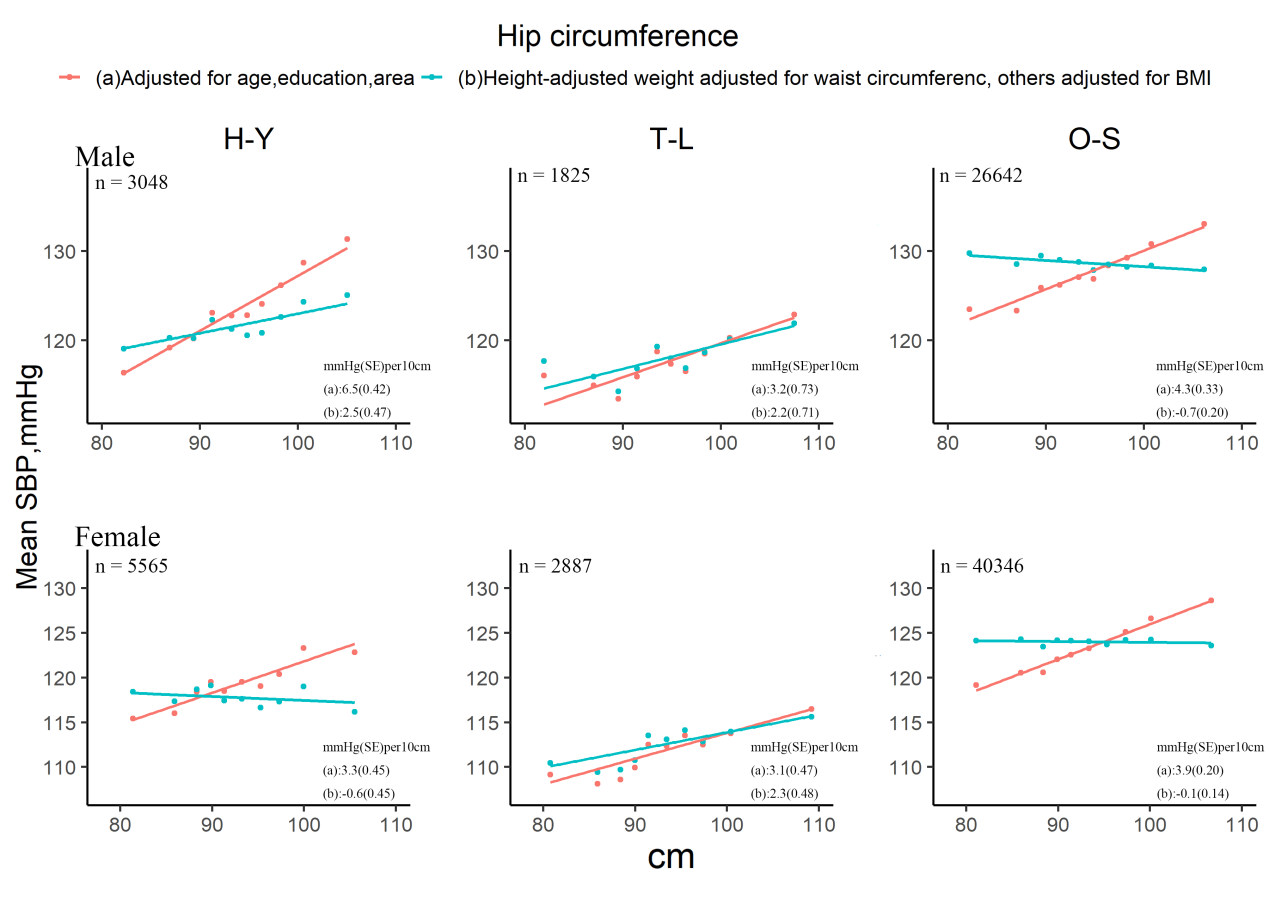


Figure S4. Sex-specific associations between HC and SBP for the three populations, before and after adjustment for BMI.

H-Y: Han ethnic in Yunnan, T-L: Tibetans in Lhasa, O-S: other seven ethics


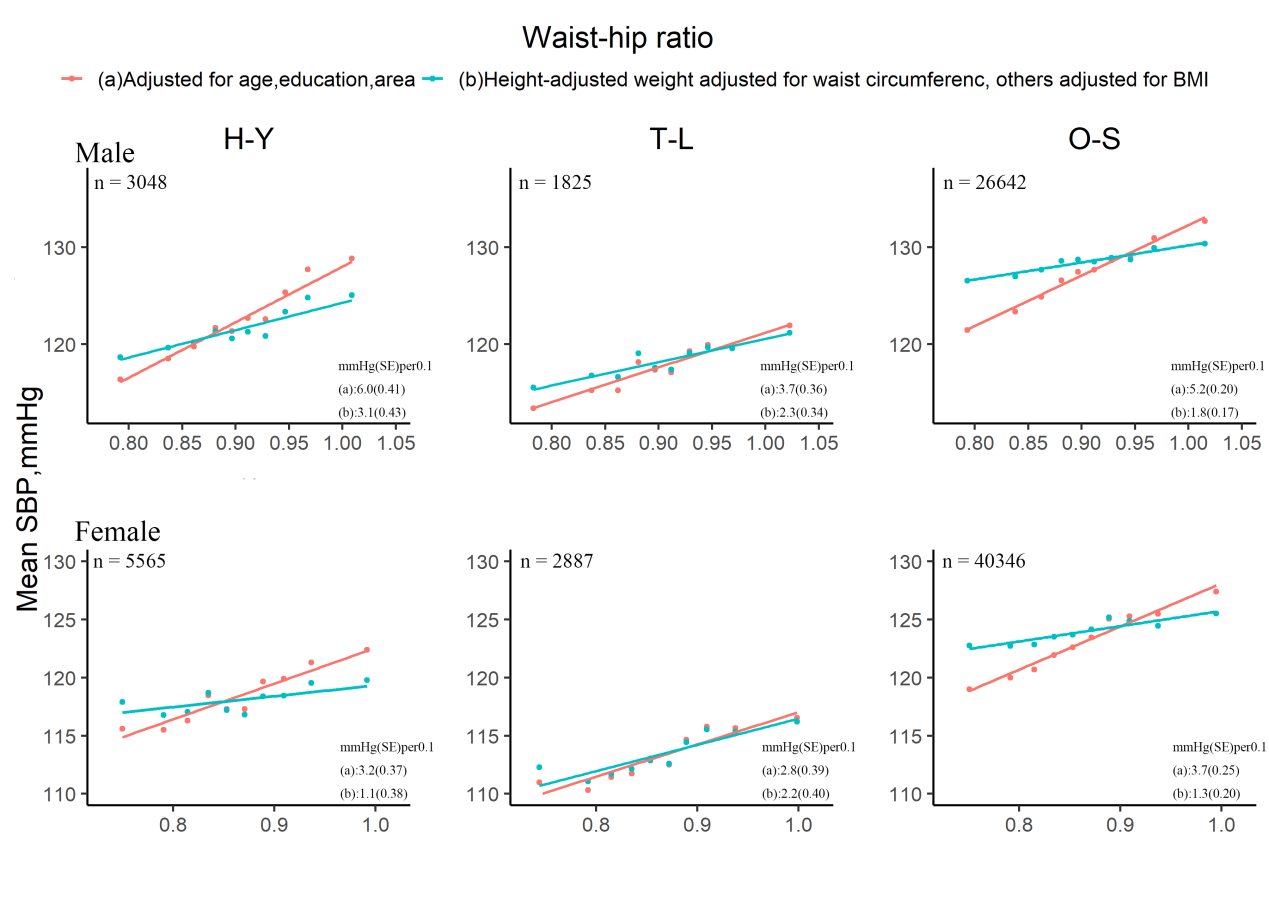


Figure S5. Sex-specific associations between WHR and SBP for the three populations, before and after adjustment for BMI.

H-Y: Han ethnic in Yunnan, T-L: Tibetans in Lhasa, O-S: other seven ethics


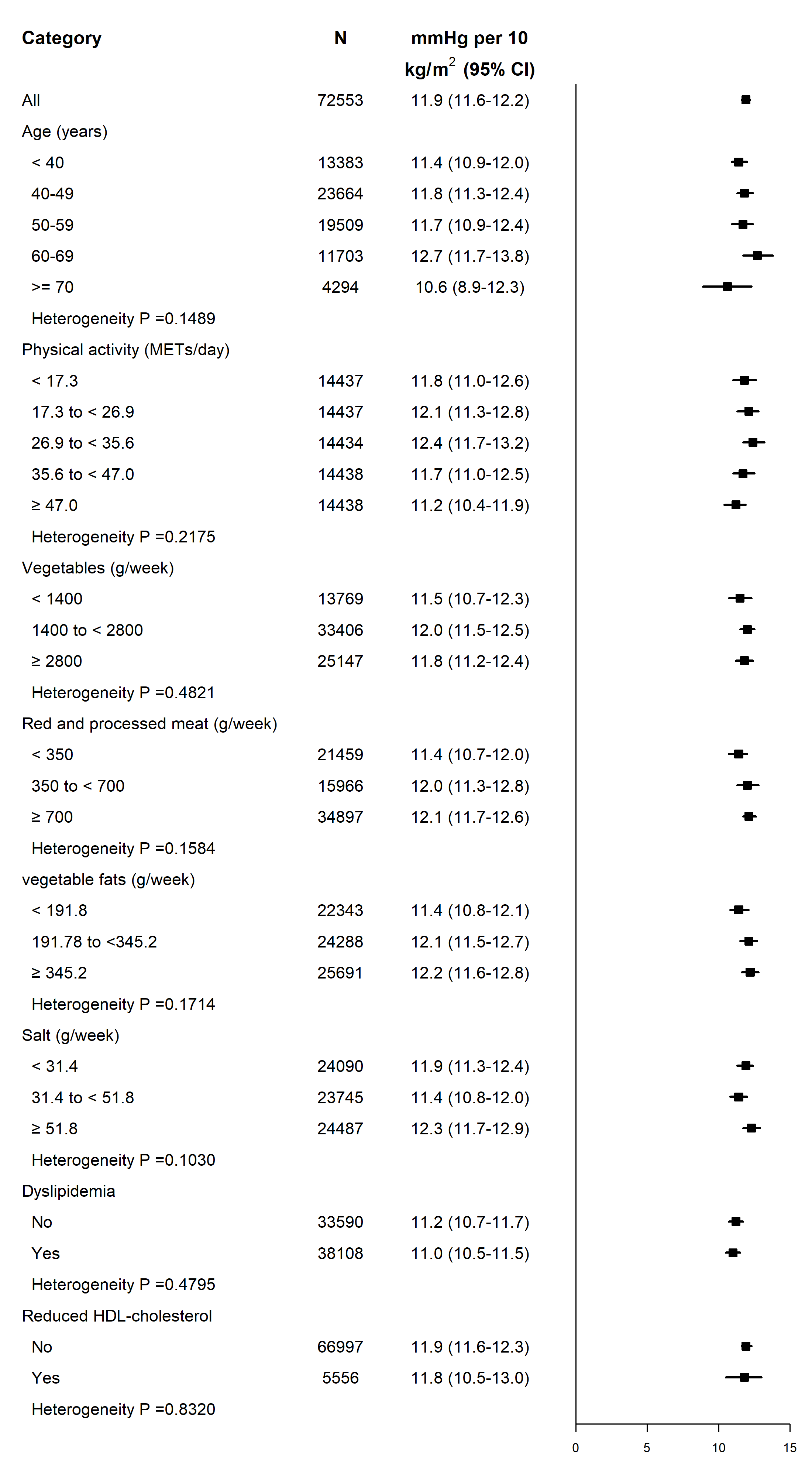
Figure S6. Higher SBP for each 10 kg/m^2^ BMI increase in different subgroups (continued)


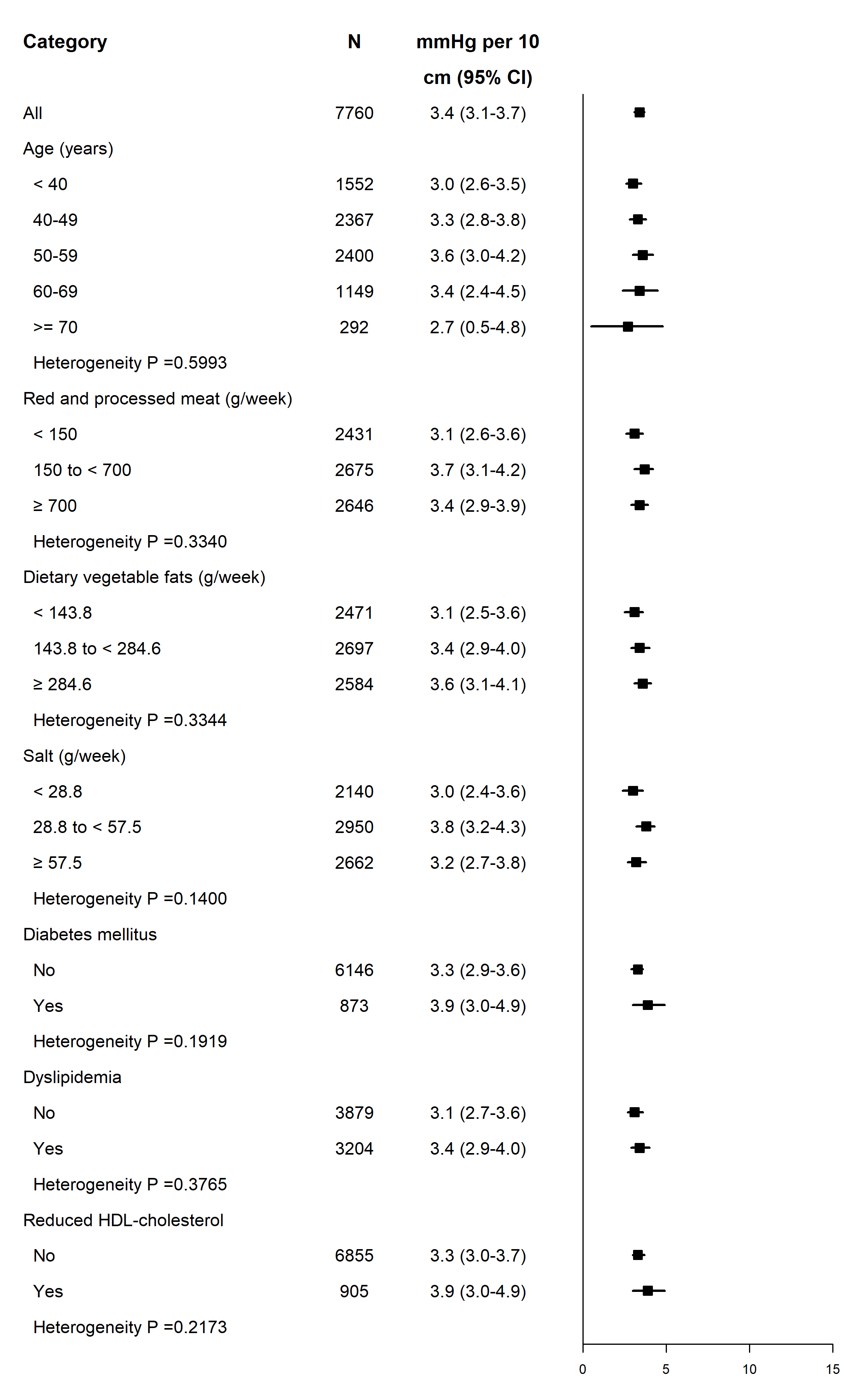
Figure S7. Higher SBP for each 10 cm WC increase in different subgroups (continued)
